# Heritable Burden of Community Sudden Death by Autopsy and Molecular Phenotyping for Precision Genotype Correlation

**DOI:** 10.1016/j.jacep.2024.10.027

**Published:** 2024-12-18

**Authors:** Zian H. Tseng, James W. Salazar, Julianne Wojciak, W. Patrick Devine, Brielle A. Kinkead, Matthew Yee, David Eik, Jean Feng, Andrew J. Connolly, Ellen Moffatt

**Affiliations:** a Section of Cardiac Electrophysiology, Division of Cardiology, Department of Medicine, University of California-San Francisco, San Francisco, California, USA; b Cardiovascular Genetics Center, University of California-San Francisco, San Francisco, California, USA; c Department of Medicine, University of California-San Francisco, San Francisco, California, USA; d Department of Pathology, University of California-San Francisco, San Francisco, California, USA; e School of Medicine, University of California-San Francisco, San Francisco, California, USA; f Department of Epidemiology and Biostatistics, University of California-San Francisco, San Francisco, California, USA; g Office of the Chief Medical Examiner, City and County of San Francisco, San Francisco, California, USA

**Keywords:** autopsy, genetics, sudden cardiac death

## Abstract

**BACKGROUND:**

Sudden cardiac death (SCD) genetic studies neglect the majority occurring in older decedents with cardiovascular pathology.

**OBJECTIVES:**

This study sought to determine the burden of genetic disease in unselected adult sudden deaths by precision genotype–postmortem phenotype correlation.

**METHODS:**

The authors used autopsy, histology, and toxicology to adjudicate cause and identify high-suspicion phenotypes (eg, hypertrophic cardiomyopathy) among presumed SCDs aged 18 to 90 years referred to the county medical examiner from February 2011 to January 2018. They tested 231 genes associated with sudden death and correlated genotype with postmortem phenotypes, including myocardial analysis. Family history in high-suspicion phenotype cases was obtained.

**RESULTS:**

Of 856 autopsied presumed SCDs, families of 359 consented and 306 cases (66% cardiac cause) ultimately underwent genetic testing (mean age 62 years, 74% male). Seventy-five cases met high-suspicion phenotype criteria (8.8%), of which 36 underwent testing; 18 families met with a genetic counselor. We found 14 cases with autosomal dominant or X-linked pathogenic/likely pathogenic (P/LP) variants (apparent yield 4.6%); 6 had concordant cause (corrected yield 2%). Yields restricted to autopsy-confirmed cardiac causes (2.5%) and high-suspicion phenotypes (2.7%) were similar. Myocardial genotyping in 14 high-suspicion decedents matched negative blood genotyping, thus did not support somatic mosaicism. Myocardial RNA in a P/LP *PKP2* carrier without phenotype demonstrated nonsense-mediated escape as potential mechanism for incomplete penetrance. One-half of high-suspicion cases had a family history of a related condition or sudden death.

**CONCLUSIONS:**

In this 7-year countywide study, 2% of total sudden deaths and 2.5% of confirmed SCDs had identifiable genetic cause, corrected for genotype–phenotype concordance. These results do not support routine genetic testing for community sudden deaths, particularly without autopsy.

Determining the burden of sudden cardiac death (SCD) due to heritable cardiovascular disease is necessary to identify affected individuals and family members at elevated genetic risk for timely screening, diagnosis, and risk stratification *before* SCD.^1,2^ Prior work in SCD genetics has concentrated on selected referral populations (ie, young, autopsy-negative, “normal heart” primary electrical diseases) enriched for genetic yield,^3–6^ but which represent a fraction of the total SCD burden.

Notably, these studies exclude older decedents and decedents with common cardiovascular pathology, such as coronary artery disease (CAD) and cardiomyopathy, which collectively underlie >50% of autopsy-confirmed causes of sudden death due to arrhythmia.^7^ Yet these prevalent cases of SCD in the community are the chief focus of prevention efforts such as primary prevention implantable cardioverter-defibrillators (ICDs), and may have distinct genetic causes (eg, familial hypercholesterolemia leading to early sudden death due to CAD).^8–10^ Conversely, population-level genetic studies of community SCD may have been negative despite thousands of cases^11^ due to a nonspecific phenotype definition that includes up to 40% due to noncardiac causes.^7^ Even postmortem genetic SCD studies have not consistently excluded all noncardiac causes such as occult overdose, which requires toxicology to detect,^12,13^ and do not evaluate tissue-level phenotyping to enhance genotype–phenotype correlation. Thus, genetic findings in autopsy-based studies of SCD may have reported “false-positives” or “false-negatives” due to incompletely investigated phenotypes, and further evidence is needed to support the expanding practice of clinical genetic testing in decedents and survivors of sudden cardiac arrest.^14^

Therefore, we sought to determine the total burden of underlying genetic cause in unselected adult sudden deaths, refined by autopsy to precise postmortem phenotypes in the San Francisco POST SCD (Postmortem Systematic Investigation of SCD)^7^ by: 1) testing a comprehensive panel of genes associated with monogenic cardiovascular (eg, long-QT syndrome, arrhythmogenic right ventricular cardiomyopathy) and noncardiovascular (eg, sudden unexplained death in epilepsy [SUDEP]) sudden death conditions; 2) assessing genotype correlation with postmortem phenotypes including myocardial genotyping and expression studies to evaluate mechanisms (eg, somatic mosaicism) for blood genotype-phenotype discordance; and 3) family screening and counseling of decedents with high-suspicion cardiovascular phenotypes (eg, premature CAD, hypertrophic cardiomyopathy) to assess burden of heritable disease beyond that captured by genetic testing.

## METHODS

### INCLUSION CRITERIA AND SELECTION OF CASES FOR POSTMORTEM INVESTIGATION.

Detailed methods of POST SCD have been previously described.^7,15^ Briefly, POST SCD is a prospective countywide autopsy study of consecutive out-of-hospital cardiac arrest (OHCA) deaths attributed to World Health Organization (WHO)-defined SCD, that is, presumed SCDs per new nomenclature,^1^ among adults aged 18 to 90 years in San Francisco County. OHCAs were defined using CARES (Cardiac Arrest Registry to Enhance Survival) criteria.^16^

We included OHCA deaths if the death was witnessed, sudden, and unexpected, and if the person was last seen alive and symptom-free within 24 hours before unwitnessed death (WHO-defined SCD, hereafter “presumed SCD”).^17^ Resuscitated persons who survived to hospital admission were considered cardiac arrest survivors and excluded.^18^ Deaths were excluded if they had a terminal illness, end-stage renal disease on hemodialysis, an alternative identifiable noncardiac cause of sudden death before autopsy (eg, scene evidence of drug use or suicide), or admission within 30 days for noncardiac illness or surgical procedure.

The initial POST SCD cohort (February 1, 2011, to March 1, 2014) captured every presumed SCD reported to the Medical Examiner countywide and comprised 525 cases. In the extended cohort (March 1, 2014, to January 1, 2018), An additional 331 cases were included by a random selection method to mitigate bias (all incident cases every third day based on medical examiner call schedule). Inclusion and exclusion criteria were the same as the original study period.

### POSTMORTEM INVESTIGATION AND ADJUDICATION OF CAUSES OF DEATH.

A complete postmortem examination was performed by a board-certified forensic pathologist including detailed heart and brain examinations and vitreous chemical analyses (Supplemental Appendix, Supplemental Methods).^7,15^ Toxicology screening was performed for all persons aged ≤75 years and for persons aged >75 years without obvious cause of death at autopsy. A multidisciplinary committee reviewed comprehensive premortem records and postmortem findings to adjudicate: 1) whether the OHCA death met WHO criteria for presumed SCD; 2) the underlying cause of death; and 3) whether presumed SCD was due to arrhythmic cause potentially rescuable with ICD. Specifically, sudden death due to arrhythmia was a presumed SCD for which no identifiable noncardiac or cardiac/non-arrhythmic cause (eg, tamponade, pump failure) was found. Occult overdose as cause of sudden death was defined by levels of drugs or substances exceeding published toxicologic thresholds.^19,20^

### GENETIC TESTING AND VARIANTS OF UNCERTAIN SIGNIFICANCE ANALYSIS.

For cases with consent for genetic testing and an adequate autopsy specimen (predominantly peripheral blood and rarely, left ventricular [LV] tissue), we performed exome sequencing. For a pilot cohort (n = 19), a commercial panel of 143 genes (Supplemental Appendix, Supplemental Table 1) associated with primary arrhythmia and cardiomyopathy disorders, and 6 genes associated with SUDEP was used. For the remaining 287 cases, a panel of 231 genes (Supplemental Appendix, Supplemental Table 1) associated with cardiovascular (eg, lipid disorders, arrhythmia, cardiomyopathy; 215 genes) and non-cardiovascular disorders (eg, SUDEP, thrombophilia; 16 genes) implicated in sudden death was filtered from the exome sequencing data. Pathogenic (P), likely pathogenic (LP), and variants of uncertain significance (VUS) were adjudicated according to American College of Medical Genetics criteria. Identified variants were reviewed by an expanded team including a molecular pathologist and certified genetic counselor (CGC) to determine whether the variant was concordant with postmortem phenotype and attributable to the cause of death. For inherited lipid disorders, concordance was defined by CAD on autopsy.

In an exploratory analysis to query potentially significant VUSs, we employed variant- and gene-level evaluations to query potentially significant VUSs. On the variant level, we further investigated VUSs with ≤3 heterozygotes in the genome aggregation database (gnomAD v3.1.2) reference population and predicted to be deleterious using in silico tools (Combined Annotation Dependent Depletion [CADD] score ≥30).^21,22^ On the gene level, we compared rare variant frequencies for genes with >3 missense variants in the POST SCD cohort to those in gnomAD to assess potential enrichment. Rare variants were defined as missense variants that had ≤20 total alleles in gnomAD and were not classified as benign or likely benign by ClinVar. The expanded multidisciplinary committee then reviewed the total evidence for potential role and concordance of VUSs meeting rarity and CADD score thresholds with postmortem phenotype and cause of sudden death.

### FAMILIAL SCREENING IN DECEDENTS WITH HIGH-SUSPICION PHENOTYPES.

Given the limited sensitivity of genetic testing, we implemented a family screening protocol for decedents with high-suspicion phenotypes for which guidelines advise familial screening.^2,9,23–27^ The high-suspicion phenotypes included primary electrical disease, arrhythmogenic right ventricular cardiomyopathy (ARVC), hypertrophic cardiomyopathy (HCM), bicuspid aortic valve, and the following conditions for decedents <50 years old: CAD, nonischemic dilated cardiomyopathy (DCM), and thoracic aortic aneurysm and dissection. Decedents of occult overdose were excluded from family screening since other postmortem findings were incidental.

Next-of-kin for decedents with high-suspicion phenotypes were offered a complimentary CGC session for interested family members. During the session, the CGC took a pedigree (≥3 generations when available) focusing on cardiovascular disease and sudden death with medical record review attempted for all diagnoses and sudden deaths. When genetic testing was performed, the results and implications were explained. Family risk assessment was performed per published guidelines, and family members were advised of screening recommendations with referrals provided as indicated.^2^

### MYOCARDIAL TISSUE GENOTYPING FOR HIGH-SUSPICION PHENOTYPES WITH NEGATIVE BLOOD GENOTYPING.

Standard genetic testing of peripheral blood may miss genetic disease caused by somatic mosaicism in the affected tissue.^28^ Thus, we performed whole exome sequencing on DNA extracted directly from LV tissue sampled at time of sudden death for patients with a high-suspicion cardiac autopsy phenotype (including ARVC, HCM, primary electrical disease, and premature DCM) where no culprit variant was identified in peripheral blood testing, to evaluate somatic mosaicism as potential genetic cause of death.

### MYOCARDIAL TISSUE EXPRESSION ANALYSIS FOR P/LP VARIANT CARRIER WITHOUT PHENOTYPE.

For a decedent with a truncating P/LP *PKP2* variant and arrhythmic death, but without pathologic evidence of ARVC, we hypothesized that escape of nonsense-mediated decay may be a mechanism for incomplete penetrance. Briefly, we isolated RNA from right ventricular [RV] tissue and performed reverse transcription. Allele-specific expression of the wildtype and truncating variants was quantified using droplet digital polymerase chain reaction probes (detailed methods can be found in the Supplemental Appendix, Supplemental Methods).

### STATISTICAL ANALYSIS.

Study population characteristics were summarized using means with SDs, 95% CIs, and proportions. For between-group comparisons, we used *t*-test and chi-square as appropriate. A 2-tailed *P* < 0.05 was considered statistically significant. For multiple hypothesis testing of variant allele frequency, *P* values were corrected by the Benjamini–Hochberg method to determine false discovery rate–corrected Q values, which were considered significant when Q was <0.05.

## RESULTS

### STUDY POPULATION.

**FIGURE 1:**
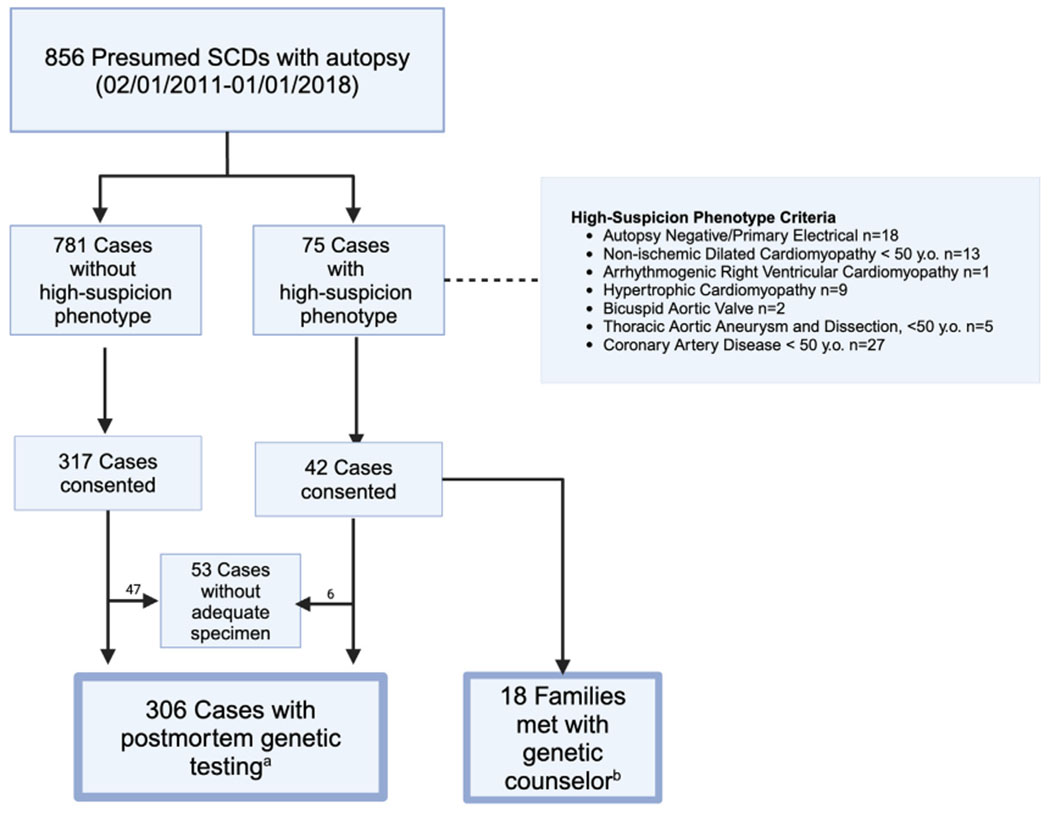
Genetic Testing and Family Screening in the San Francisco POST SCD Study From February 1, 2011, to January 1, 2018 *World Health Organization definition of sudden cardiac death (SCD) (ie, presumed SCD) was employed: sudden and unexpected death within 1 hour of new symptom onset (witnessed), or within 24 hours of having been observed alive and symptom free (unwitnessed). ^a^Of 306 cases that underwent genetic testing, 36 met high-suspicion phenotype criteria. ^b^Of 18 high-suspicion cases that met with a genetic counselor, 17 had genetic testing. POST SCD = Postmortem Systematic Investigation of SCD study; y.o. = years old.

Between February 1, 2011, and January 1, 2018, 856 presumed SCDs were autopsied in San Francisco County. Families of 359 (42%) consented and 306 (36%) cases ultimately underwent genetic testing; autopsy identified 75 high-suspicion phenotype cases (8.8% of all presumed SCDs) for family screening (Figure 1). Thirty-six of the 75 high-suspicion phenotype cases consented and received genetic testing, representing 11.8% of the tested cohort.

The tested cohort (mean age 62 years, 74% men) reflected the diverse population of San Francisco County (Supplemental Appendix, Supplemental Table 2).^29^ Of the 306 tested cases, 62% were adjudicated as sudden deaths due to arrhythmia (Table 1, Central Illustration), 34% had noncardiac causes, and 4% cardiac, nonarrhythmic causes (eg, heart failure, tamponade) of sudden death. The leading cardiac causes were CAD (35%) and cardiomyopathy (13%), whereas the leading noncardiac causes were occult overdose (11%) and sudden neurologic death (7%).

### COUNTYWIDE BURDEN OF SUDDEN DEATH WITH UNDERLYING GENETIC CAUSE.

Using our sudden death gene panel, we identified 34 P/LP variants in 33 of 306 cases (10.8%); 19 decedents had P/LP variants with autosomal-recessive inheritance, all heterozygous, thus representing unaffected carrier status. The remaining 15 P/LP variants in 14 decedents were either X-linked (n = 1, *GLA*) or autosomal-dominant (AD) inheritance (Table 2). Seven of these 15 P/LP variants had a concordant autopsy phenotype, which in 6 of 7 cases also represented the cause of sudden death. The exception was a decedent with DCM on autopsy and a concordant P/LP *TNNT2* variant but whose unrelated cause of sudden death was occult overdose. In the 6 cases of P/LP variants concordant with phenotype and cause of sudden death, 5 were arrhythmic and 1 nonarrhythmic. The majority of detected P/LP variants (8 of 15, 53%) had no concordance with autopsy phenotype (Table 2).

In summary, 14 of 306 presumed SCDs were found to have an AD or X-linked P/LP variant—an apparent yield of 4.6%—but only 6 P/LP variants were concordant with a phenotype that had caused sudden death, therefore a corrected yield of 2%. Excluding cases with noncardiac and nonarrhythmic causes of sudden death by autopsy, the corresponding corrected yields were 2.5% (5 of 201) and 2.6% (5 of 190) for autopsy-confirmed SCDs and arrhythmic deaths, respectively. Thus, countywide over the 7-year study period, only 2% of all sudden deaths, 2.5% of SCDs, and 2.6% of arrhythmic deaths had an identified genetic cause (Central Illustration). Restricting the denominator to the 36 high-suspicion phenotype cases, yield was similar (2.7%, 1 of 36), and slightly higher in the 48 decedents ≤45 years old (4.2%, 2 of 48). In no instance did genetic testing modify the cause of sudden death adjudicated by autopsy alone.

### ANALYSIS OF UNCERTAIN VARIANTS.

We identified 644 total variants—the 34 aforementioned P/LP variants and 610 VUSs—in 170 of 245 tested genes and 265 of 306 cases (79%). The mean ± SD number of variants identified in arrhythmic (2.2 ± 1.6) and non-arrhythmic sudden deaths (2.0 ± 1.5) was similar (*P* = 0.5). Corrected for multiple hypothesis testing, the rare variant allele frequency was similar among the arrhythmic deaths (n = 190) compared to gnomAD for all genes with >3 variant alleles detected in our cohort (Supplemental Appendix, Supplemental Table 3). Of the 610 VUSs, 30 (4.9%) met criteria for rarity (≤3 heterozygotes in gnomAD v3.1.2) and predicted deleteriousness (≥30 CADD score) for further investigation of potential genotype–phenotype correlation. Of these, we found 5 variants with possible concordance, all with DCM phenotype (1 *LMNA*, 4 *TTN*). However, we determined insufficient evidence to conclude clinical significance for these variants.

### MYOCARDIAL GENOTYPING AND EXPRESSION STUDIES FOR CASES WITH APPARENT GENOTYPE/PHENOTYPE DISCORDANCE.

Fourteen decedents had high-suspicion phenotypes involving a disorder of the myocardium (6 premature DCM, 4 primary electrical disease, 3 HCM, 1 ARVC) but negative blood genetic testing. We further evaluated these cases for somatic mosaicism by testing DNA extracted from myocardial tissue. The exome sequencing of DNA from LV tissue (RV for the ARVC case) demonstrated no variants distinct from blood testing and thus, no evidence for somatic mosaicism as an explanation for negative genetic testing despite high-suspicion phenotype (Central Illustration).

For cases with a P/LP variant without associated cardiac phenotype, we extracted ventricular RNA to evaluate for biased allele-specific expression as a mechanism for incomplete penetrance; only 1 case with P/LP *PKP2* variant but no gross or histologic evidence of ARVC phenotype had adequate RV RNA for further assessment. Since the variant was predicted to result in early truncation of the translated protein, the mRNA transcript was anticipated to undergo nonsense-mediated decay. To test whether escape of nonsense-mediated decay may explain incomplete penetrance in this patient, and more generally in other patients with pathogenic *PKP2* variants, we quantified RNA expression of wildtype and P/LP *PKP2* alleles with the expectation that expression of the truncating allele would be low relative to wildtype allele. RNA extracted from RV revealed 68.9% of expression corresponded to the wildtype allele and 31.1% to the P/LP allele (Central Illustration).

### FAMILY SCREENING OF CASES WITH HIGH-SUSPICION PHENOTYPES.

**FIGURE 2:**
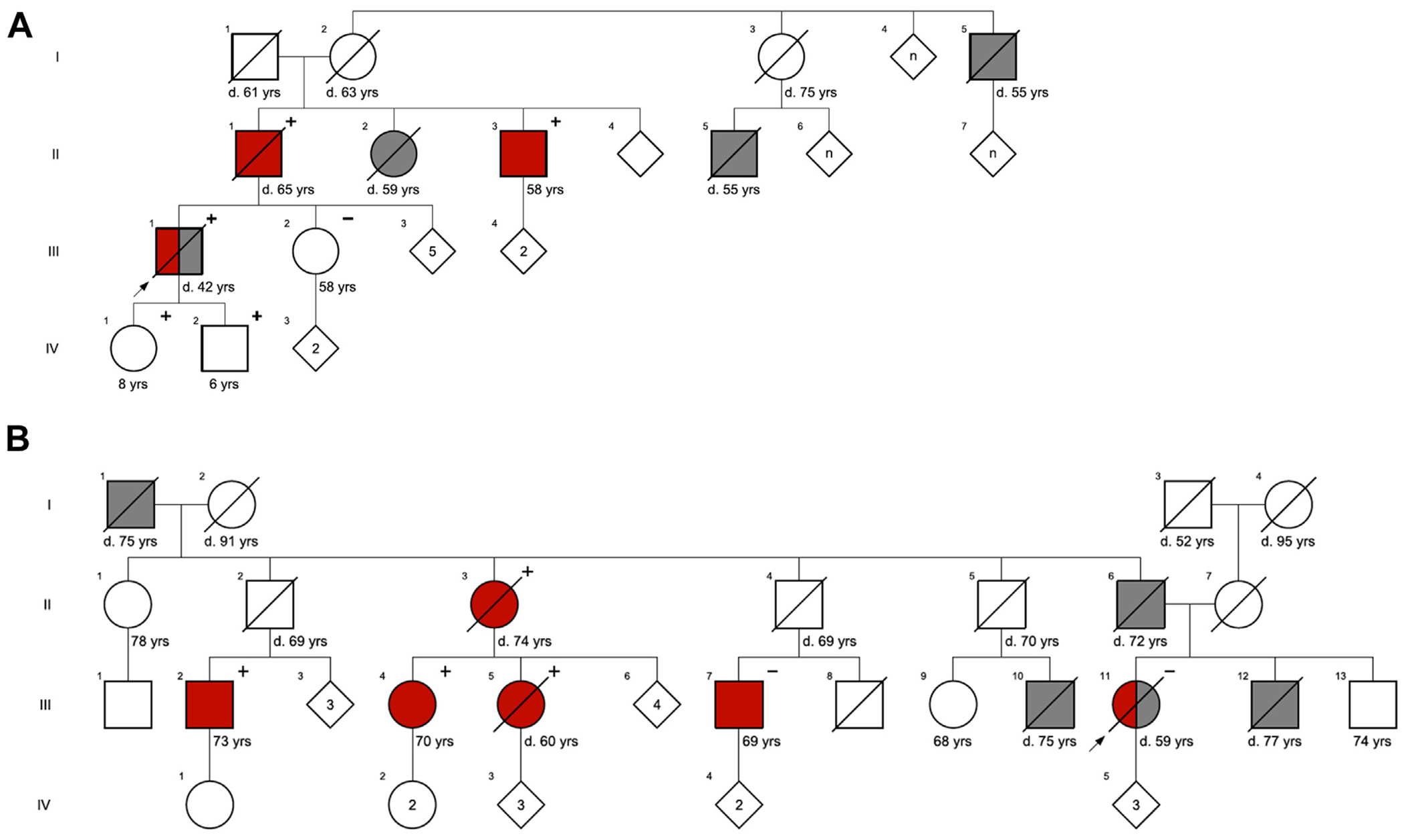
Pedigrees of HCM Cases Established With a Certified Genetic Counselor (A) Four-generation pedigree of 42-year-old male (III-1) who died of sudden arrhythmic death due to hypertrophic cardiomyopathy (HCM) with concordant pathogenic *MYBPC3* variant with multiple family members affected by HCM (with same genotype) or sudden death. The father and uncle of the proband received implantable cardioverter-defibrillators (ICDs) with the latter subsequently receiving an appropriate ICD shock for ventricular fibrillation. (B) Four-generation pedigree of 59-year-old female (III-11) who died of sudden arrhythmic death and was found to have HCM with no pathogenic mutations detected. Patient had multiple relatives affected by HCM some with a concordant genotype, 3 of which carried a likely pathogenic mutation *MYH7* that was not detected in the proband nor a first cousin with a clinical diagnosis of HCM. Based on the pedigree, a second variant was suspected to contribute to familial HCM. Family screening led to the implantation of 3 implantable cardioverter-defibrillators in this family. Symbols: Arrow = proband, squares = male sex, circles = female sex, diamonds = multiple individuals of both male and female sex, + = pathogenic variant present, − = pathogenic variant absent. Symbols that are filled are affected with red = hypertrophic cardiomyopathy and gray = sudden death. The diagonal line through a symbol indicates the person is deceased.

Seventy-five of 856 (8.8%) total presumed SCDs met prespecified high-suspicion autopsy phenotypes (Figure 1). Families of 18 cases met with the study CGC (17 with adequate specimens and genetic testing in the proband); we found a positive family history of the same/related condition in 6 (33%), sudden death in 8 (44%), and either in 10 (56%) (Table 3), whereas only 1 of the tested high-suspicion probands had a P/LP variant (1 of 36, 2.7%). Meeting with the study CGC led to referral for primary prevention ICDs in family members of 2 decedents, both with HCM, and ultimately led to successful rescue from ventricular fibrillation in 1 family member (Figure 2).

## DISCUSSION

In this 7-year study of the genetics underlying 306 unselected adult sudden deaths in an entire metropolitan area (66% with cardiac cause, 34% noncardiac by autopsy), a comprehensive panel of genes associated with sudden death revealed a variant responsible for the sudden death in only 6 decedents (2%). The yield remained low when restricted to only autopsy-confirmed sudden cardiac deaths (2.5%) or arrhythmic deaths (2.6%), despite myocardial genotyping for evaluation of somatic mosaicism in high-suspicion phenotype cases. The majority (53%) of P/LP variants with AD or X-linked inheritance did not have concordant postmortem phenotype, and we did not find evidence to upgrade any detected VUS to P/LP. These data extend current guidance, based on young or autopsy-negative sudden deaths, that genetic testing is not advisable in sudden deaths without careful phenotyping^2,30^ to older sudden deaths missed by those studies.

As clinical genetic testing becomes more ubiquitous, these findings lend critical insight into its utility in adult sudden deaths and their surviving family members^30^—a clinical population often referred for genetic testing. The unique nature of the POST SCD study facilitated rigorous evaluation of genotype–phenotype correlation including molecular phenotyping with myocardial genotyping and expression studies. Compared with prior studies in *selected* sudden death populations (eg, young individuals) that found P/LP variants in 10% to 25% of cases,^3–5^ in this unselected sudden death population, the yield of a P/LP variant concordant with cause of sudden death, that is, “true positives,” was low at 2% and remained low at 2.5% when restricted to autopsy-defined SCD and despite sequencing ventricular myocardium for possible hidden P/LP variants (ie, somatic mosaicism). In contrast to younger or “autopsy-negative” sudden death cohorts enriched for rare cardiac or arrhythmogenic disorders, our cohort revealed a distinct, underappreciated profile of heritable sudden death in the general population, including more indolent conditions such as transthyretin cardiac amyloidosis and familial hypercholesterolemia leading to early CAD. Among the unselected sudden deaths 18 to 45 years in our study, corrected yield of genetic testing was 4.2%—still substantially lower than yields reported in selected referral cohorts of the same age range (13%-27%).^3,4^ Notably, despite testing fewer noncardiovascular genes, we detected a P/LP variant associated with antithrombin-III deficiency in a 43-year-old man with fatal pulmonary embolism. This suggests a potential role for phenotype-directed genetic testing for heritable noncardiac causes of sudden death when confirmed by autopsy. Overall, the corrected yield of genetic testing in our cohort, though modest, represents the real-world signal of sudden death with genetic causes in the community, with ramifications for family members of victims. Indeed, family counseling in our study averted sudden death in a surviving family member.

Critical lessons can also be gained from the “false positives” in our study. In a sudden death cohort, it was unexpected that the majority (53%) of detected P/LP variants had no evidence of their associated conditions despite histologic evaluation for subtle manifestation. To further investigate genotype-positive cases with phenotype discordance, we extended phenotyping to myocardial expression studies in a truncating *PKP2 P/LP* variant carrier without ARVC phenotype and found evidence for escape of nonsense mediated decay to overcome haploinsufficiency as a mechanism for incomplete penetrance. This in-human demonstration may support gene therapeutic approaches to restore *PKP2* RNA levels to circumvent ARVC as tested in animal models.^31^

Our inclusion of sudden deaths due to occult overdose as detected on toxicology further demonstrates the potential for incorrect attribution of genetic findings—as in the decedent with DCM and a phenotype-concordant P/LP *TNNT2* variant but who died of occult overdose. The increasingly appreciated burden of sudden deaths due to occult overdose^13,32^ likely contaminates the interpretation of genetic testing in “autopsy-negative” cases and studies without toxicology,^12^ and suggests caution in interpreting genetic studies of presumed sudden cardiac arrest and SCD confounded by noncardiac causes.^11^ In sum, genetic studies that define sudden death cases without comprehensive postmortem methods, that is, presumed SCDs, likely contain a substantial proportion of false positives.^33^

Prior sudden death studies of similar sample size have identified VUS candidates for reclassification to P/LP.^4^ In an exploratory analysis, we did not find any VUSs with sufficient evidence to support pathogenicity and after excluding nonarrhythmic deaths, we found no difference in rare variant allele frequency compared with the reference gnomAD population database. Although these data do not exclude other undetected monogenic or polygenic risk in our cohort, they suggest caution in interpreting genome wide association studies of variants that include common and rare VUSs and a phenotype definition that presumes cardiac cause of arrest or sudden death without autopsy.^11^

Our family screening protocol of decedents with high-suspicion cardiovascular phenotypes, 10% of the overall cohort, revealed familial disease or sudden death in 56%, whereas only 1 had positive genetic testing. This disparity indicates deficiencies in the current understanding of genetic causes of sudden death. Thus, contemporary genetic testing alone may be insufficient to detect familial risk, which also includes shared lifestyle and environmental factors, and a “phenotype-first” approach to familial screening may represent a higher yield strategy for sudden death prevention.

### STUDY LIMITATIONS.

The intentional inclusion of noncardiac sudden deaths and noncardiac genes dilutes the overall yield. However, even excluding noncardiac deaths, the corrected yield of concordant P/LP mutations was largely unchanged. The tested cases represent a subset of overall sudden deaths, and 6% of cases were tested using a more limited pilot panel of genes. However, our pilot panel analyzed more genes, and our overall consent rate is similar to that of genetic studies in unselected *young* sudden death cohorts that inform clinical practice.^3^ Finally this diverse, urban cohort may not generalize to other populations. However, the prevailing causes of sudden death we found are consistent with established causes (eg, CAD and cardiomyopathy) in the general population.

## CONCLUSIONS

The contribution of genetic cause to adult sudden deaths in the community is modest even with rigorous tissue examination for phenotype and excluding noncardiac deaths. Our results suggest that postmortem genetic testing of all sudden death cases should be performed only after carefully establishing cause and phenotype by comprehensive postmortem investigation. Phenotype-guided screening of families of victims may yield more clinically actionable findings and enhance detection of potentially heritable disease than genetic testing alone.

## Supplementary Material

Supplement

## Figures and Tables

**CENTRAL ILLUSTRATION:**
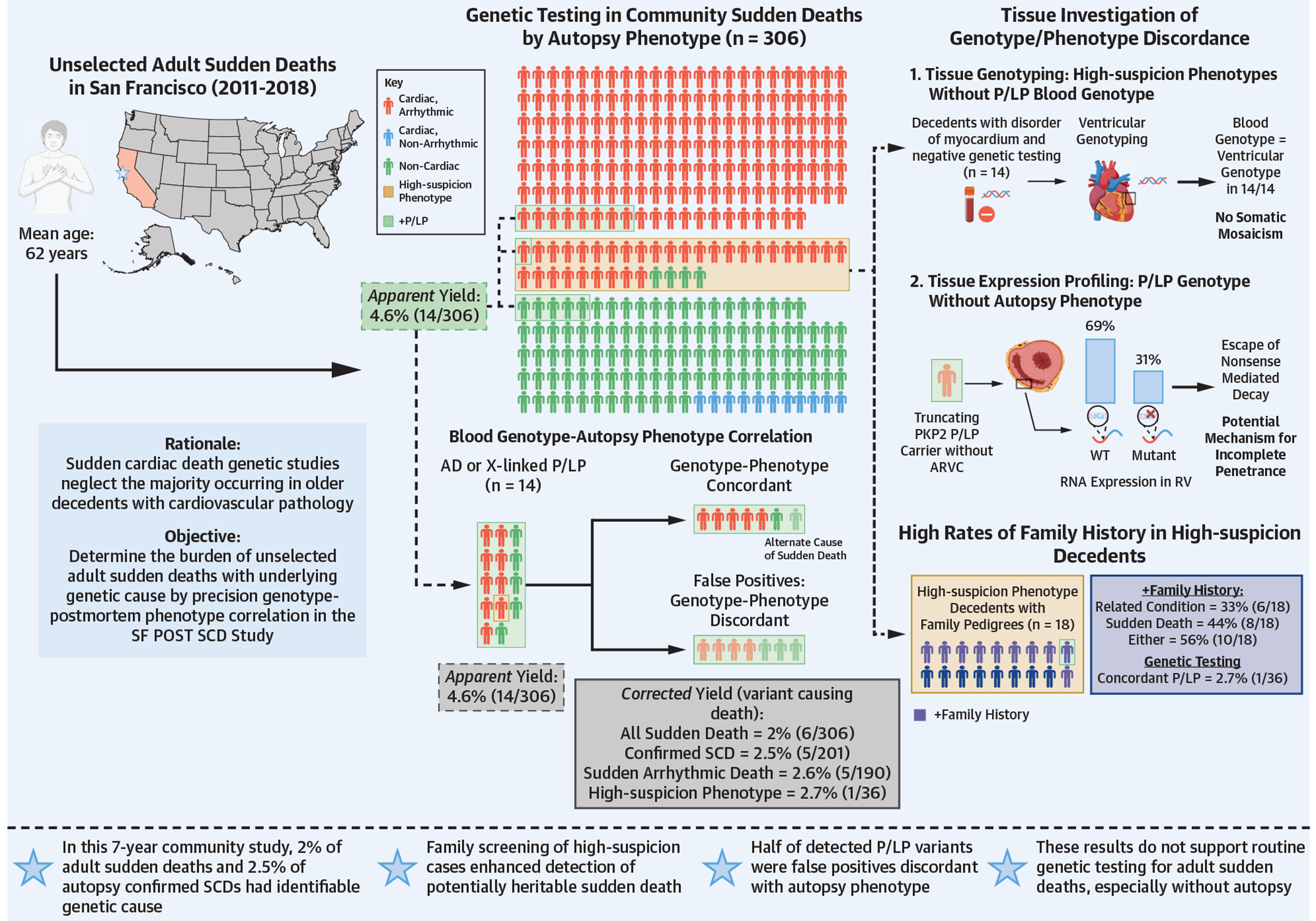
Heritable Burden of Community Sudden Death by Autopsy and Molecular Phenotyping for Precision Genotype Correlation Autopsy phenotype: Of the 306 cases that were autopsied and underwent genetic testing, 62% had arrhythmic cause (eg, coronary artery disease, cardiomyopathy), 34% noncardiac cause (eg, occult overdose, intracranial hemorrhage), and 4% cardiac, nonarrhythmic cause (eg, heart failure, tamponade) of sudden death. High-suspicion phenotypes indicated by dashed box included premature coronary artery disease, hypertrophic cardiomyopathy, and primary electric disease, among others. Color denotes cause of death. Highlighting represents cases that were found to have an autosomal dominant or X-linked pathogenic likely/pathogenic (P/LP) variant. Genotype–autopsy correlation: Blood genotype–autopsy phenotype correlation with autopsy phenotype and cause of death. Apparent yield is the percent of cases with positive genetic testing irrespective of autopsy phenotype. Corrected yield is percent of cases with a P/LP variant concordant with cause of sudden death. Tissue investigation of genotype/phenotype discordance: Myocardial genotyping and expression studies to deeply evaluate genotype-phenotype correlation in cases with apparent genotype/phenotype discordance. AD = autosomal-dominant; ARVC = arrhythmogenic right ventricular cardiomyopathy; RV = right ventricle; SCD = sudden cardiac death; SF POST SCD = San Francisco Postmortem Systematic Investigation of SCD study; WT = wildtype.

**TABLE 1:** Causes of Presumed Sudden Cardiac Deaths as Determined by Autopsy (N = 306)

Arrhythmic cause	190 (62)
Acute CAD	31 (10)
Chronic CAD/prior MI	78 (25)
Cardiomyopathy	39 (13)
Hypertrophy	31 (10)
Primary electrical disease	6 (2)
Valvular disease	4 (1)
Myocarditis	1 (<1)

Cardiac, nonarrhythmic cause	11 (4)
Acute CAD with myocardial rupture or pump failure	6 (2)
Chronic CAD/prior MI with pump failure	2 (<1)
Heart failure	2 (<1)
Valvular disease	1 (<1)

Noncardiac	105 (34)
Infection	10 (3)
Neurological	20 (7)
Vascular damage	12 (4)
Occult overdose	33 (11)
Pulmonary embolism	11 (4)
Other pulmonary	4 (1)
Other noncardiac	15 (5)

Values are n (%). Other noncardiac causes (n = 15) included asphyxia (n = 2), bowel obstruction (n = 1), cancer (n = 1), gastrointestinal hemorrhage (n = 2), hernia and associated complications (n = 3), hyperglycemia or diabetic ketoacidosis (n = 2), hypoglycemia (n = 2), hypothermia (n = 1), and peritonitis (n = 1). Other pulmonary (n = 4) included asthma (n = 1), end-stage chronic obstructive pulmonary disease (n = 2), and obesity hypoventilation syndrome (n = 1).

CAD = coronary artery disease; MI = myocardial infarction.

**TABLE 2:** Cases With Autosomal Dominant or X-Linked P/LP Variants and Postmortem Phenotype (n = 14)^a^

Age, y	Sex	Race/Ethnicity	Gene	Variant Location^b^	Associated Disorder	Mechanism of Death	Cause of Death	Postmortem Phenotype
Concordant with autopsy phenotype and cause of death (n = 6)								
61	M	White	*LDLR*	c.682G>T p.Glu228*	Familial hypercholesterolemia	Arrhythmic	Chronic CAD	Chronic CAD
65	M	Hispanic	*SCN5A*	c.3010_3022delTGCATTGCCACCC p.Cys1004fs	Brugada, long QT Syndrome, cardiac conduction disease, DCM	Arrhythmic	Hypertensive heart disease	Arrhythmia in setting of hypertensive heart disease
86	F	Black	*TTR*	c.424G>A [homozygous] p.Val142Ile	TTR amyloid	Arrhythmic	Amyloidosis	Cardiac amyloidosis
42	M	Asian	*MYBPC3*	c.2905C>T p.Gln969*	HCM, DCM	Arrhythmic	HCM	HCM
56	M	White	*TTN*	C.104413C>T p.Arg34805*	DCM	Arrhythmic	DCM	DCM
43	M	White	*SERPINC*	c.1273C>T p.Arg425Cys	Antithrombin-III deficiency	Nonarrhythmic	Pulmonary embolus	Pulmonary embolus

Concordant with autopsy phenotype but not cause of death (n = 1)								
67	M	White	*TNNT2*	c.887G>A p.Trp296*	HCM, DCM	Nonarrhythmic	Occult OD	DCM

No identified concordance with autopsy phenotype or cause of death (n = 8)								
54	M	White	*GLA*	c.869T>C p.Met290Thr	Fabry disease/HCM	Arrhythmic	Bicuspid aortic valve	No evidence of Fabry disease
52	M	Hispanic	*DEPDC5*	c.1701_1704dupAGAC p.Ser569fs	Epilepsy/SUDEP	Arrhythmic	Acute CAD	No known seizure history
42	M	Asian	*APOB*	c.10579C>T p.Arg3527Trp	Familial hypercholesterolemia	Arrhythmic	HCM	No evidence of CAD
60	F	Black	*TTR*	c.424G>A p.Val142Ile	TTR amyloid	Arrhythmic	Acute MI	No evidence of cardiac amyloidosis
48	M	White	*PKP2*	c.1613G>A p.Trp538*	ARVC, DCM	Arrhythmic	Chronic CAD	No evidence of ARVC
52	M	White	*LDLR*	c.502G>A p.Asp168Asn	Familial hypercholesterolemia	Nonarrhythmic	SUDEP	No evidence of CAD
25	M	Black	*ALPK3*	c.3526G>T p.Glu1176*	HCM	Nonarrhythmic	Occult OD	No evidence of HCM
71	M	White	*DSG2*	c.523+2T>C	ARVC	Nonarrhythmic	Aortic dissection	No evidence of ARVC

Asterisk (*) denotes a premature termination codon in the caption.

a There were 15 pathogenic/likely pathogenic (P/LP) variants in 14 decedents. The 42-year-old Asian male featured 2 P/LP variants (*MYBPC3* and *APOB*).

b All variants were heterozygous unless otherwise specified. All variants were categorized as pathogenic except for *ALPK3* and *GLA*, which were likely pathogenic.

ARVC = arrhythmogenic right ventricular cardiomyopathy; CAD = coronary artery disease; DCM = dilated cardiomyopathy; HCM = hypertrophic cardiomyopathy; OD = overdose; TTR = transthyretin.

**TABLE 3:** Family History in Sudden Deaths Meeting High-Suspicion Phenotype Criteria

Case	Decedent High-Suspicion Phenotype	Concordant P/LP Mutation^a^	Generations in Pedigree	Family History of Same/Related Condition	Family History of Sudden Death
42F, White	ARVC	No	1	No	Yes
42M, Asian	HCM	Yes, *MYBPC3*	6	Yes	Yes
59F, White	HCM	No	4	Yes	Yes
35M, White	Premature CAD	No	1	No	No
41M, White	Premature CAD	No	3	Yes	No
44M, White	Premature CAD	No	1	No	Yes
44M, White	Premature CAD	No	1	No	No
46M, White	Premature CAD	No	4	No	No
48M, White	Premature CAD	No	4	Yes	Yes
48M, Asian	Premature CAD	No	3	Yes	Yes
49M, Asian	Premature CAD	—	3	No	Yes
26M, White	Premature dilated NICM	No	4	No	No
36M, White	Primary electrical disease	No	4	No	No
38M, White	Primary electrical disease	No	4	Yes	No
37M, Black	Primary electrical disease	No	5	No	Yes
28M, Asian	Primary electrical disease	No	1	No	No
45M, White	Thoracic aortic aneurysm and dissection	No	4	No	No
48M, White	Thoracic aortic aneurysm and dissection	No	1	No	No
	**Yield of Genetic Testing**	1 (5.9%)^b^	**Yield of Family History**	6 (33%) 10 (56%)	8 (44%)

a One patient did not have an adequate specimen for genetic testing.

b A total of 36 high-suspicion phenotype cases underwent genetic testing without any other P/LP mutations for an overall yield of 2.7%.

NICM = nonischemic cardiomyopathy; other abbreviations as in Table 2.

## PERSPECTIVES

### COMPETENCY IN MEDICAL KNOWLEDGE:

In this 7-year study of 306 unselected adult sudden deaths, refined to arrhythmic and nonarrhythmic causes by autopsy, testing of 231 genes commonly associated with sudden death revealed a genetic cause in six decedents (2%). Yield remained low when restricted to autopsy-defined sudden cardiac deaths (2.5%) or high-suspicion (eg, hypertrophic cardiomyopathy) phenotypes (2.7%). Genetic causes of community sudden deaths include underappreciated, indolent conditions such as transthyretin cardiac amyloidosis and familial hypercholesterolemia, and noncardiac conditions such as thrombophilia causing fatal pulmonary embolism. These data extend current guidance that genetic testing is not advisable in sudden deaths without careful phenotyping, based on young or autopsy-negative sudden deaths, to older sudden deaths missed by those studies.

### TRANSLATIONAL OUTLOOK:

The majority (53%) of detected P/LP variants did not have concordant phenotype after comprehensive postmortem evaluation including autopsy and tissue phenotyping. Extending phenotyping to tissue molecular analyses may provide further mechanistic insights, as demonstrated in the case of a P/LP *PKP2* carrier but no autopsy or histology of evidence arrhythmogenic right ventricular cardiomyopathy, who had myocardial RNA evidence of nonsense-mediated escape as potential mechanism for incomplete penetrance.

## ABBREVIATIONS AND ACRONYMS

AD: autosomal-dominant
ARVC: arrhythmogenic right ventricular cardiomyopathy
CAD: coronary artery disease
CADD: Combined Annotation Dependent Depletion
CGC: certified genetic counselor
DCM: dilated cardiomyopathy
HCM: hypertrophic cardiomyopathy
ICD: implantable cardioverter-defibrillator
LV: left ventricular
OHCA: out-of-hospital cardiac arrest
P/LP: pathogenic/likely pathogenic
RV: right ventricular
SCD: sudden cardiac death
SUDEP: sudden unexplained death in epilepsy
VUS: variant of uncertain significance
WHO: World Health Organization
