# Supplementary material for: Heritable Burden of Community Sudden Death by Autopsy and Molecular Phenotyping for Precision Genotype Correlation
Source: JACC Clin Electrophysiol. Author manuscript; Available in PMC 2025 May 28. (PMC12117619; doi:10.1016/j.jacep.2024.10.027)
Supplement: Supplement [file NIHMS2079015-supplement-Supplement.docx]

**Supplemental Appendix**

Table of Contents

- Supplemental Methods
  - Autopsy Procedure
  - Supplemental Table 1. Genes included in Testing
  - Rare Variant calling procedure
  - Myocardial Tissue Expression Analysis
- Supplemental Results
  - Supplemental Table 2. Demographic characteristics of the study and reference populations
  - Supplemental Table 3. Rare missense variant allele frequency in sudden arrhythmic deaths compared to gnomAD

Supplemental Methods:

Autopsy Procedures

A comprehensive examination of the internal organs of the thorax, abdomen, and cranial vault was performed according to established autopsy methods.^1,2^

Cardiac Examination

A standardized, extensive examination of the heart was performed. Each heart was weighed (cardiac mass) and indexed to body mass index (cardiac mass index = cardiac mass/BMI). Orthogonal dimensions of the atria and ventricles were recorded. Valves were examined for bicuspid aortic valve, aortic leaflet perforation, evidence of endocarditis and severe aortic leaflet fibrosis/calcification that could indicate aortic stenosis. The thickness of the compact myocardium in the left ventricle (LV) was measured in 4 standard locations: septum 1 cm beneath the aortic valve, as well as posterobasal, lateral, and mid-anterior free wall. Right ventricular (RV) free wall thickness was also measured.

The epicardial surface of the heart was examined and the major extramural coronary arteries were evaluated for coronary abnormalities. Each coronary artery (left main [LM], left anterior descending [LAD], left circumflex [LCx], right coronary [RCA]) was cut in cross section every 5 mm to demonstrate narrowed segments; calcification necessitated removal of the intact arteries for fixation and chemical decalcification before cross 5 sectioning. All segments with thrombi, significant atherosclerotic plaque or evidence of dissection were sampled for histology. The narrowest segment of each coronary artery was also sampled.

The apical half of the heart was then cut in short axis cross section to yield four to five 1-cm thick rings of ventricular muscle, and the remaining heart (at the base) is cut to open each chamber along the lines of blood flow. Each ring of ventricular myocardium was examined for presence of acute myocardial infarct (MI) or scar denoting healed MI, and extent of infarction. For hearts with evidence of MI, scar dimensions were measured and classified as subendocardial, transmural, or epicardial. Full thickness sections were taken from regions of MI and across the boundary between scar and grossly normal myocardium. Finally, internally at the aortic valve, the positions of the coronary ostia at the sinuses of Valsalva were inspected for possible malformation.

Active (acute) coronary lesions were defined by the presence of a disrupted coronary plaque (tear/erosion of the luminal fibrous cap with extravasation of blood into a lipid core), luminal acute thrombus (collections of platelets, fibrin, and trapped erythrocytes/white blood cells), or both (luminal thrombus in the area of a ruptured plaque).^3^ Hearts demonstrating histologic evidence of acute MI with or without a corresponding acute coronary lesion were considered to have acute MI.

Chronic significant coronary artery disease was defined by an inactive lesion with ≥ 50% stenosis (equivalent to luminal cross-sectional area reduction ≥ 75%) without plaque disruption and thrombus in at least one major coronary artery (LM, LAD, LCx, or RCA).^3^

Findings of coronary artery bypass graft (CABG) of at least one vessel, gross or histologic evidence of acute or healed MI, acute coronary lesion, and/or chronic significant coronary artery disease (CAD) were considered CAD causes of sudden death.

Cardiac hypertrophy (without CAD, DCM or HCM) was defined as LV compact wall thickness > 1.5 cm, or cardiac mass greater than predicted for body mass index.^1^

Histological Examination

Samples of myocardium for histology were taken from 5 standard locations: septum, posterobasal, lateral, mid-anterior left ventricular free wall, and right ventricular free wall. An extra section of the high septum was also taken for histologic examination of the conduction system. Histologic sections were stained with hematoxylin and eosin and trichrome and independently examined by two pathologists (the second and fifth authors). Each cardiac slide section was examined for diagnostic criteria for the following:

Myocarditis:

Diagnosis based on the presence of an inflammatory infiltrate of the myocardium with necrosis and/or degeneration of adjacent myocytes not typical of the ischemic damage associated with CAD.^4^ The presence of a polymorphous inflammatory infiltrate including eosinophils, with or without myocyte necrosis, was considered evidence of hypersensitivity reaction involving the heart.

Hypertrophic cardiomyopathy (HCM): Presence of (1) concentric LV hypertrophy (nondilated cavity) in the absence of another cardiac or systemic condition that could lead to comparable hypertrophy, (2) LV septal to free wall ratio of greater than 1.3 for disproportionate septal hypertrophy (the usual form of HCM), or (3) myofiber disarray in the upper LV septum, along with thick-walled slit-lumen intramyocardial arteries.^5,6^

Hypertrophy (without CAD, DCM or HCM): Hypertrophic cardiomyocytes without myofiber disarray.

Non-Ischemic/Dilated CM: Increased cardiac mass and LV dimensions without evidence of CAD, valvular heart disease, pericardial disease, chronic hypertension or congenital heart malformation.

Arrhythmogenic right ventricular dysplasia/cardiomyopathy (ARVD): Presence of transmural fibrofatty infiltration of the RV myocardium.^7,8^

Amyloidosis: Presence of widespread interstitial myocardial and/or vascular deposits of amorphous protein with characteristic birefringence when stained with Congo red and viewed with polarized light.^9^

Sarcoidosis: Presence of noncaseating granulomas in ventricular myocardium.^10^

Examination of specialized cardiac conduction system: In cases where no other cardiac pathology was identifiable, examination of the cardiac conduction system was performed. The specialized tissues of the SA node and AV conduction axis were dissected en bloc using well-established landmarks, according to standard methods.^11,12^

Assessment of Myocardial Fibrosis: Fibrosis was quantified by digital image analysis of Masson’s trichrome stained sections using Aperio ImageScope software’s Positive Pixel Count algorithm calibrated for hue and color saturation thresholds. Fibrosis scores were calculated as the sum total pixel count for subendocardial, perivascular/interstitial, and replacement fibrosis as a percentage of total slide tissue area for all sections.

Postmortem vitreous chemistries (electrolytes, creatinine, urea nitrogen, and glucose) were obtained for all subjects.

**Supplemental Table 1. Genes included in Testing**

| Category | Genes* |
| --- | --- |
| **Cardiovascular Conditions** | |
| Arrhythmia, Cardiomyopathy | **Both:** ANK2 CACNA1C CACNA2D1 CACNB2 CALM1 CALM2 CALM3 CASQ2^r^ GJA5 HCN4 KCND3 KCNE1 KCNE2 KCNH2 KCNJ2 KCNQ1 RYR2 SCN10A SCN5A SNTA1 TRDN^r^ AKAP9 GPD1L KCNE3 KCNE5^x^ KCNJ5 KCNJ8 MYL4 RANGRF SCN1B SCN2B SCN3B SCN4B TRPM4 CAV3 FKRP^r^ FKTN^r^ EYA4 GATAD1 ABCC9 ACTC1 ACTN2 ANKRD1 BAG3 CSRP3 DES DOLK^r^ DSC2 DSG2 DSP FLNC JPH2 JUP LAMA4 LDB3 LMNA MYBPC3 MYH7 MYL2 MYL3 MYLK2 NEBL NEXN PKP2 PLN PRKAG2 RBM20 TMEM43 TNNC1 TNNI3 TNNT2 TPM1 TTN VCL CALR3 CTF1 CTNNA3 DTNA FHL2 ILK LRRC10 MYH6 MYOM1 MYOZ2 MYPN PDLIM3 PLEKHM2 PRDM16 TCAP TMPO BRAF DMD^x^ DNAJC19^r^ ELAC2^r^ EMD^x^ FHL1^x^ GAA^r^ GLA^x^ HRAS KRAS LAMP2^x^ MAP2K1 MAP2K2 MTO1^r^ NF1 NRAS PTPN11 RAF1 RASA1 RIT1 RRAS SDHA^r^ SGCD SHOC2 SLC22A5^r^ SOS1 SOS2 SPRED1 TAZ^x^ TXNRD2  **Expanded Panel Only:** TECRL^r^ HEY2 GNB5 ALPK3^r^ TNNI3K ACTA1 CDH2 MIB1 MYLK3 MYO6 ANO5^r^ CAPN3^r^ HNRNPDL LZTR1 MRAS MYOT PNPLA2^r^ PPA2 PPP1CB SLC25A4 SYNE1 SYNE2 TOR1AIP1  **Pilot Panel Only:** CHRM2 A2ML1 ACADVL AGL ALMS1 CBL CPT2 CRYAB SLMAP TMEM70 |
| Congenital Heart Disease | **Both:** GATA4^r^ GATA6 NKX2-5  **Expanded Panel Only:** GATA5 TBX20 MED12^x^ TBX5  **Pilot Panel Only:** N/A |
| Hereditary Hemorrhagic Telangiectasia | **Expanded Panel Only:** ACVRL1 ENG EPHB4 SMAD4  **Pilot Panel Only:** N/A |
| Lipid | **Both:** APOB LDLR LDLRAP1^r^ PCSK9  **Expanded Panel Only:** ABCG5^r^ ABCG8^r^ APOA1 APOA5 APOC2^r^ APOC3 CYP27A1^r^ GPIHBP1^r^ LIPA^r^ LMF1 LPL SCARB1 ANGPTL3^r^ CETP CREB3L3 CYP7A1^r^ GCKR LIPC^r^ LRP6 ABCA1 GPD1^r^ LCAT^r^ MTTP^r^ SAR1B  **Pilot Panel Only:** N/A |
| Pulmonary arterial hypertension | **Both:** KCNK3 KCNA5^r^  **Expanded Panel Only:** ATP13A3 BMPR2 CAV1 EIF2AK4^r^ GDF2 SMAD9 AQP1 TBX4 |
| Thoracic aortic aneurysm and dissection | **Both:** TGFB3  **Expanded Panel Only:** ACTA2 EFEMP2^r^ FOXE3 LOX MAT2A MFAP5 MYH11 MYLK PRKG1 SMAD3 TGFB2 TGFBR1 TGFBR2 SLC2A10^r^ SMAD2 BGN^x^ COL3A1 FBN1 FLNA^x^ PLOD1^r^ |
| Miscellaneous | **Both:** TTR - Amyloid  **Expanded Panel Only:** NOTCH1 - Bicuspid Aortic Valve |
| **Noncardiovascular Conditions** | |
| Sudden Unexpected Death in Epilepsy | **Expanded Panel Only:** CSTB^r^ DEPDC5 HCN2 KCNA1 PRRT2 SCN1A SCN2A SCN8A CACNA1A  **Pilot Panel Only:** KCNQ2 KCNQ3 KCNT1 PCDH19^x^ SCN9A SLC2A1 |
| Thrombophilia | **Expanded Panel Only:** F2 F5 MPL PROC PROS1 SERPINC1 |
| Cerebral autosomal dominant arteriopathy with subcortical infarcts and leukoencephalopathy (CADASIL) | **Expanded Panel Only:** HTRA1 |

*Unless otherwise denoted, mode of inheritance for gene is dominant.

^r^ Mode of inheritance for gene is recessive.

^x^ Mode of inheritance for gene is X-linked.

**Myocardial Tissue Expression Analysis Methods**

**Genomic DNA Extraction and Quantification**

Genomic DNA (gDNA) from the blood, left ventricle, and right ventricle were isolated using the Zymo Quick-DNA/RNA Miniprep kit. The concentration and integrity of isolated gDNA was determined on an Agilent Bioanalyzer.

**RNA isolation and reverse transcription**

Total RNA was isolated from a small fraction of right ventricle muscular tissue (approx. 0.1cm3) using the Qiagen RNEasy Mini Kit. RNA concentration and integrity were determined using Nanodrop 2000 Spectrophotometer. Reverse transcription of 160ng of RNA was completed with the High-Capacity cDNA Reverse Transcription Kit (ThermoFisher Scientific) according to manufacturer protocol.

**Droplet Digital PCR**

Each droplet digital PCP (ddPCR) reaction consisted of 12.5 uL of ddPCR Supermix for Probes (no dUTP) (BioRad), 50 ng of DNA, 1.25 ul primer/probe, and nuclease-free water up to 25 ul. Each sample was run in triplicate. Droplets were generated using autoDG droplet generator (BioRad) by combining 20 uL of the reaction mixture with 70 uL of Droplet Generation Oil for Probes (BioRad). PCR amplification was performed using BioRad T100 Thermocycler using the following protocol: 1) 95 °C for 10 min; (2) 94 °C for 30 s; (3) 58 °C for 1 min; (4) steps 2; and 3 repeat 39 times; (5) 98 °C for 10 min; (6) hold at 4 °C. The fluorescent intensity of each droplet was measured using the BioRad Droplet Reader Qx200 and analysis of ddPCR data was performed using QX Manager Standard Edition Version 2.1.0.25.

**Supplemental Table 2. Demographic characteristics of the study and reference populations***

| **Characteristic** | **Sudden Cardiac Deaths with Autopsy and Genetic Testing† (N = 306)** | **Sudden Cardiac Deaths with Autopsy** ‡  **(N = 856)** | **Adult Population, San Francisco County 2014  (N = 717,884)** **§** | **Genome Aggregation Database**  **(N = 141,456)** |
| --- | --- | --- | --- | --- |
| **Age – years** | | | | |
| Mean | 62.0 ± 15.0 | 60.2 ± 14.0 | 45.4 ±17.8 |  |
| Range | 25-89 | 18-89 | 18-90 |  |
| **Male sex -- no. (%)** | 226 (74) | 620 (72) | 364,799 (51) | 76,702 (54) |
| **Race or ethnic group -- no. (%)** | | | | |
| Asian | 51 (17) | 165 (19) | 236,902 (33) | 25,285 (18) |
| Black | 37 (12) | 140 (16) | 43,073 (6) | 12,487 (9) |
| Hispanic or Latino | 16 (5) | 62 (7) | 107,682 (15) | 17,720 (12) |
| Other | 2 (1) | 24 (3) | 35,894 (6) | 3,614 (3) |
| White | 200 (65) | 465 (54) | 294,332 (41) | 82,350 (58) |

*Plus–minus values are means ±SD.

†Included are sudden cardiac deaths from February 1, 2011, to January 1, 2018

that underwent autopsy and genetic testing

‡ Included are sudden cardiac deaths from February 1, 2011, to January 1, 2018

that underwent autopsy

§ Data are from the American Community Survey.^7^

**Supplemental Table 3. Rare missense variant allele frequency in sudden arrhythmic deaths compared to gnomAD.***

| Gene | Variant Allele Frequency | | Original P-value | Q-value |
| --- | --- | --- | --- | --- |
|  | **Sudden Arrhythmic Deaths** | **gnomAD** |  |  |
| KCNH2 | 0.016 | 0.005 | 0.02 | 0.31 |
| HCN4 | 0.013 | 0.005 | 0.04 | 0.31 |
| SCN1A | 0.013 | 0.005 | 0.04 | 0.31 |
| MYH11 | 0.030 | 0.009 | 0.04 | 0.31 |
| CTNNA3 | 0.011 | 0.004 | 0.08 | 0.40 |
| VCL | 0.011 | 0.004 | 0.08 | 0.40 |
| DEPDC5 | 0.013 | 0.007 | 0.12 | 0.48 |
| SYNE1 | 0.026 | 0.032 | 0.13 | 0.48 |
| LAMA4 | 0.008 | 0.004 | 0.16 | 0.52 |
| SCN10A | 0.018 | 0.011 | 0.21 | 0.54 |
| ANK2 | 0.008 | 0.017 | 0.23 | 0.54 |
| SYNE2 | 0.022 | 0.026 | 0.23 | 0.54 |
| MYH6 | 0.013 | 0.008 | 0.25 | 0.54 |
| TNNI3K | 0.013 | 0.005 | 0.25 | 0.54 |
| ALPK3 | 0.026 | 0.011 | 0.29 | 0.57 |
| CACNA1C | 0.005 | 0.012 | 0.34 | 0.57 |
| FBN1 | 0.017 | 0.008 | 0.34 | 0.57 |
| AKAP9 | 0.018 | 0.013 | 0.35 | 0.57 |
| APOB | 0.029 | 0.022 | 0.38 | 0.57 |
| MYPN | 0.005 | 0.003 | 0.38 | 0.57 |
| EPHB4 | 0.009 | 0.004 | 0.41 | 0.58 |
| PROS1 | 0.013 | 0.005 | 0.44 | 0.59 |
| SCN5A | 0.005 | 0.010 | 0.45 | 0.59 |
| CACNA1A | 0.013 | 0.007 | 0.51 | 0.62 |
| FLNC | 0.018 | 0.015 | 0.52 | 0.62 |
| RYR2 | 0.011 | 0.016 | 0.54 | 0.62 |
| CACNB2 | 0.005 | 0.004 | 0.67 | 0.74 |
| TTN | 0.174 | 0.179 | 0.84 | 0.90 |
| DSP | 0.013 | 0.014 | 1 | 1 |
| TRPM4 | 0.003 | 0.005 | 1 | 1 |

gnomAD indicates genome aggregation database.

*Variants were filtered to exclude in-frame deletions and insertions, variants classified by ClinVar as benign or likely benign, and those with more than 20 total alleles identified in the database (non-rare).

P values were corrected by the Benjamini–Hochberg method to determine false discovery rate–corrected Q values which were considered significant when Q was less than 0.05

**Bibliography**

1. Connolly A, Finkbeiner W, Ursell P, Davis R. Autopsy Pathology: A Manual and Atlas. Third. Philadelphia, PA: Elsevier;

2. Tseng ZH, Olgin JE, Vittinghoff E, et al. Prospective Countywide Surveillance and Autopsy Characterization of Sudden Cardiac Death: POST SCD Study. Circulation 2018;137(25):2689–700.

3. Farb A, Tang AL, Burke AP, Sessums L, Liang Y, Virmani R. Sudden coronary death. Frequency of active coronary lesions, inactive coronary lesions, and myocardial infarction. Circulation 1995;92(7):1701–9.

4. Aretz HT, Billingham ME, Edwards WD, et al. Myocarditis. A histopathologic definition and classification. Am J Cardiovasc Pathol 1987;1(1):3–14.

5. Maron BJ, McKenna WJ, Danielson GK, et al. American College of Cardiology/European Society of Cardiology clinical expert consensus document on hypertrophic cardiomyopathy. A report of the American College of Cardiology Foundation Task Force on Clinical Expert Consensus Documents and the European Society of Cardiology Committee for Practice Guidelines. J Am Coll Cardiol 2003;42(9):1687–713.

6. Maron BJ, Towbin JA, Thiene G, et al. Contemporary definitions and classification of the cardiomyopathies: an American Heart Association Scientific Statement from the Council on Clinical Cardiology, Heart Failure and Transplantation Committee; Quality of Care and Outcomes Research and Functional Genomics and Translational Biology Interdisciplinary Working Groups; and Council on Epidemiology and Prevention. Circulation 2006;113(14):1807–16.

7. Corrado D, Fontaine G, Marcus FI, et al. Arrhythmogenic right ventricular dysplasia/cardiomyopathy: need for an international registry. Study Group on Arrhythmogenic Right Ventricular Dysplasia/Cardiomyopathy of the Working Groups on Myocardial and Pericardial Disease and Arrhythmias of the European Society of Cardiology and of the Scientific Council on Cardiomyopathies of the World Heart Federation. Circulation 2000;101(11):E101-106.

8. McKenna WJ, Beiras AC, Lado MP. The cardiomyopathies. Br Heart J 1994;72(6 Suppl):S1.

9. Falk RH. Diagnosis and management of the cardiac amyloidoses. Circulation 2005;112(13):2047–60.

10. Kim JS, Judson MA, Donnino R, et al. Cardiac sarcoidosis. Am Heart J 2009;157(1):9–21.

11. Virmani R, Ursell PC, Fenoglio JJ. Examination of the heart. Hum Pathol 1987;18(5):432–40.

12. Lev M, Widran J, Erickson EE. A method for the histopathologic study of the atrioventricular node, bundle, and branches. AMA Arch Pathol 1951;52(1):73–83.

13. American Community Survey. Washington, DC: U.S. Census Bureau; 2014.
